# The use of clamping grips and friction pads by tree frogs for climbing curved surfaces

**DOI:** 10.1098/rspb.2016.2867

**Published:** 2017-02-22

**Authors:** Thomas Endlein, Aihong Ji, Shanshan Yuan, Iain Hill, Huan Wang, W. Jon P. Barnes, Zhendong Dai, Metin Sitti

**Affiliations:** 1 Max Planck Institute for Intelligent Systems, Heisenbergstrasse 3, 70569 Stuttgart, Germany; 2 Institute of Bio-inspired Structure and Surface Engineering, Nanjing University of Aeronautics and Astronautics, 29 Yudao Street, Nanjing 210016, People's Republic of China; 3 Centre for Cell Engineering, University of Glasgow, Joseph Black Building, University Avenue, Glasgow G12 8QQ, UK

**Keywords:** climbing, friction grip, adhesive pad, subarticular tubercle, tree frog

## Abstract

Most studies on the adhesive mechanisms of climbing animals have addressed attachment against flat surfaces, yet many animals can climb highly curved surfaces, like twigs and small branches. Here we investigated whether tree frogs use a clamping grip by recording the ground reaction forces on a cylindrical object with either a smooth or anti-adhesive, rough surface. Furthermore, we measured the contact area of fore and hindlimbs against differently sized transparent cylinders and the forces of individual pads and subarticular tubercles in restrained animals. Our study revealed that frogs use friction and normal forces of roughly a similar magnitude for holding on to cylindrical objects. When challenged with climbing a non-adhesive surface, the compressive forces between opposite legs nearly doubled, indicating a stronger clamping grip. In contrast to climbing flat surfaces, frogs increased the contact area on all limbs by engaging not just adhesive pads but also subarticular tubercles on curved surfaces. Our force measurements showed that tubercles can withstand larger shear stresses than pads. SEM images of tubercles revealed a similar structure to that of toe pads including the presence of nanopillars, though channels surrounding epithelial cells were less pronounced. The tubercles' smaller size, proximal location on the toes and shallow cells make them probably less prone to buckling and thus ideal for gripping curved surfaces.

## 1. Introduction

Tree frogs are able to climb smooth surfaces such as broad leaves or smooth rock faces by using expanded toe pads on each of their digits. Each pad adheres by secreting a watery fluid, generating capillary forces resulting from the thin fluid layer between the pads and the surface. Previous studies have investigated the details of the attachment mechanisms and the attachment performance of various tree frog species [1–4]. Although tree frogs are often found resting on broad and flat surfaces such as leaves, they have to reach the leaves by climbing smaller curved objects such as twigs and smaller branches. An obvious way is to grip around objects by using their long digits. This gripping and clamping technique relies mostly on the friction between the digits (or other body parts) and the (cylindrical) object and has been studied intensively in many arboreal animals including primates [5,6], reptiles [7,8], some insects [9,10] and robots [11].

A recent study by Herrel *et al.* [12] tested the impressive climbing ability of phyllomedusan tree frogs on very narrow substrates and could show that frogs use different sets of digits depending on the substrate's diameter. Manzano *et al.* [13] studied the detailed limb anatomy in two species of arboreal frogs, highlighting the capability and dexterity of their limbs to grasp and climb challenging terrains. Furthermore, electrostimulations of limb muscles and manually pulling the frog away from a cylindrical dowel showed that frogs are able to exert a powerful grip [13]. However, studies investigating the clamping forces in climbing frogs are otherwise absent as tree frogs have been studied mostly for the adhesive capabilities of their expanded toe pads against flat surfaces. In addition to those pads, each digit also bears subarticular tubercles which could aid in friction and/or adhesion when the digits clamp an object [14]. To the best of our knowledge, no other studies have yet addressed the function of these structures in tree frogs. Our observations on White's tree frogs (*Litoria caerulea*) have shown that these structures barely come into contact with a flat surface [15]. We propose that these structures will be more relevant when frogs clasp around objects and are mainly used for increasing the friction between the fingers and the grasped surface. Interestingly, many tree frogs have fairly long digits in comparison with the size of the palm [13,16] which would not necessarily help in adhesion but could be very important for a prehensile grip. It is therefore interesting to study the function of individual digits and the forces they can generate when tree frogs climb cylindrical objects.

In this study, we investigate how Chinese gliding frogs (*Rhacophorus dennysi*) climb and hold on to cylindrical objects by (i) measuring the ground reaction forces involved in climbing one fixed-sized cylindrical column, (ii) measuring the contact area of the adhesive pads and subarticular tubercles coming into contact when climbing differently-sized cylindrical tubes, and (iii) by comparing the maximum friction and adhesion forces generated by individual pads and tubercles in restrained animals. We ask the following questions: do tree frogs use clamping forces to climb cylindrical structures or do they rely solely on tangential friction forces to propel themselves upwards (similar to climbing a flat surface)? Is their clamping grip affected by surface roughness? How much do the subarticular tubercles come in contact with the surface when digits are wrapped around an object and do they aid the friction forces generated by the pads?

## 2. Material and methods

Our (non-invasive) experiments adhered to the Animal Behaviour Society guidelines (United Kingdom) for the use of animals in research. All data are available from the Dryad repository (http://dx.doi.org/10.5061/dryad.pd7vt) [17].

### (a) Study animals

Six individuals of the Chinese gliding frog (*Rhacophorus dennysi*) were obtained from a local supplier in China. This species was chosen for its large body size and large limb span, which would enable them to grasp around our larger diameter cylinders (see below). The animals were housed in simple terraria that contained broad-leaved indoor plants and dry branches for climbing and resting. Frogs were kept at room temperature (20–28°C) and were fed with water and crickets *ad libitum*.

Each frog was weighed to the nearest gram on a digital balance. The forelimb span (maximum left-to-right distance) was measured to the nearest millimetre by carefully stretching out the limbs along a ruler. Electronic supplementary material, table S1 lists the mass, the snout–vent length and the forelimb span for the individual frogs. For our experiments, we tried to use each frog equally often, when possible. The detailed number of repetitions per frog is given for each experiment in the data available electronically.

### (b) Force measurement set-up

**Figure 1.**
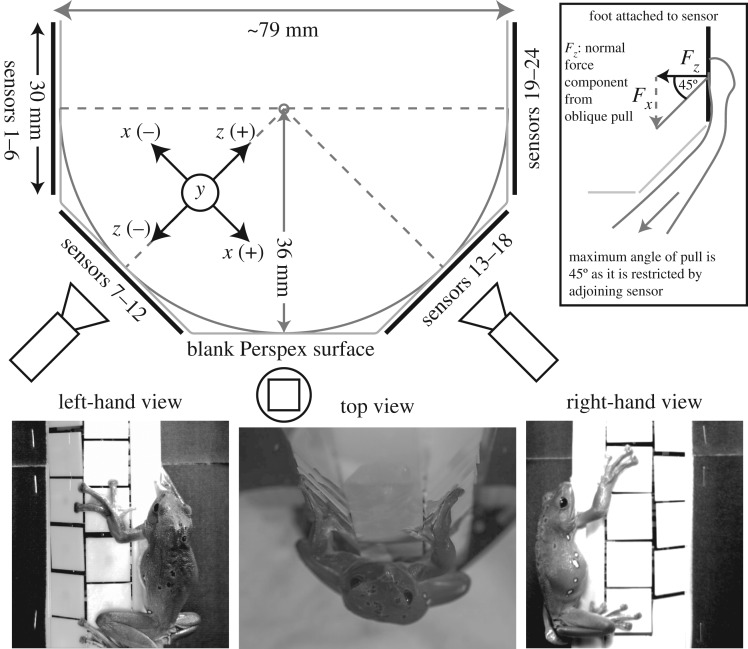
Set-up for measuring ground reaction forces. An array of 24 three-dimensional force transducers was arranged in four columns and six rows (with one blank column in the centre) to make up half of an octagon (inscribing circle with *r* = 36 mm). The normal force component (*F*_*z*_) along the *z*-axis is pointing from the centre of each sensor to the centre of the column with the tangential force (*F*_*x*_) perpendicular to it (*x*-axis). The *y*-axis was defined along the direction of gravity. Inset: owing to the arrangement of the sensors at 45° to each other, an oblique pull by the frog on the sensor can have a normal force component; therefore, only if *F_z_* > *F_x_*, can a ‘true’ clamping force be distinguished.

To measure ground reaction forces of frogs climbing a cylindrical column, 24 separate custom-built three-dimensional force transducers (similar to [18]) were arranged around the front half of an octagonally shaped tube (figure 1). The size of the tube was limited to a minimum diameter of 79 mm for the size of the component force transducers. The individual force transducer platforms (approx. 30 × 30 mm each) were arranged in four columns and six rows, where two columns were placed on the left-hand sides of the octagon and two on the right-hand sides with one row in the middle left blank. This way the forces involved in a clamping grip of opposing limbs can be obtained (see also electronic supplementary material, video S1). We defined the *x*-axis as the left–right axis of each force transducer which would resolve the lateral force component (*F*_*x*_) of a climbing frog. The *y*-axis was defined along the direction of gravity and would resolve the fore–aft components of a climbing frog. The *z*-axis was defined as the normal component, perpendicular to the surface of each transducer (*F*_*z*_) and would resolve the tensile and compressive forces.

As the hindlimbs of a vertically upwards climbing animal usually push into the surface (compressive normal forces) in order to compensate for the pivoting torque around the centre of gravity, these normal forces would mask the clamping forces; we therefore focused our force analysis on the forelimbs only. However, as the sensors were arranged around the sides of an octagon, i.e. in a 45° angle towards each other, positive normal force could also occur when a frog pulled on a sensor in an oblique way (figure 1 inset). ‘True’ clamping forces thus are only visible when the (compressive) normal forces are greater in magnitude than the lateral forces.

In addition to the force measurements, climbing animals were filmed using three synchronized high-speed video cameras filming the position of the animal from two sides (2 × Basler A602f, 695 × 465 pixels, triggered at 50 Hz) and one top view (Olympus i-Speed 3, 1280 × 1024 pixels, triggered at 100 Hz). From the different camera perspectives, we extracted the positions of the limbs placed on the corresponding force sensors.

To examine the effect of gripping force (which is dominated by compressive normal force and friction) versus attachment force (tensile normal force), we used different ‘coatings’ on the force transducer platform tiles. We used the bare platforms as a smooth surface, and the platform segments covered with a rough sandpaper (P320 from 3M, Minnesota, USA; average particle diameter 46.2 μm). Adhesive pads adhere well to smooth surfaces [16,19] but are often challenged on rough surfaces. Rough surfaces thus may promote larger compressive forces.

The anti-adhesive nature of the rough sandpaper substrate was tested by challenging the frogs to stay attached to a flat, platform covered with the sandpaper which could be tilted. By slowly rotating the platform from a horizontal into a vertical position at approx 16 ± 9 deg s^−1^ (mean ± s.d.; *N* = 17) frogs eventually slipped and detached. In only two out of 17 trials, did frogs manage to stay attached until the board reached a vertical position (90°). In all other cases, frogs detached on reaching an angle of 75 ± 6° (mean ± s.d.). This is in contrast to the attachment of the frogs to a flat smooth vertical surface, where frogs adhered without any problems.

### (c) Contact area measurements

To measure the contact area of pads and subarticular tubercles in climbing frogs we used transparent, Perspex substrates. We allowed the frogs to climb a flat sheet and two cylindrical tubes (44 mm and 120 mm diameter; see also images in figure 3) illuminated with arrays of small LEDs positioned on the top and bottom of the sheet/tubes, so that the light would be directed inwards into the Perspex material. This technique, developed from a ‘cat walk’ [20], has been used before on climbing frogs [15,16], revealing high contrast images of the bright body parts in contact against a dark background. For the cylindrical tubes, we used three synchronized high-speed video cameras (details see above) arranged in a triangular fashion around the tube in order to maximize the chance of seeing the frog's limbs centred in one view, whereas for the flat substrate a single high-speed camera was sufficient. To minimize distortion effects of the curved surface, we selected frames where the limb of concern was placed near the centre of the tube. Any cylinder substantially smaller in diameter would have not allowed us to measure the contact area accurately enough, due, in part, to optical distortions and in part to digits masking the camera's view of the area of contact.

### (d) Individual toe pad and subarticular tubercle force measurements

To measure the adhesion and friction forces on individual pads, we used a force transducer set-up similar to the one used before by [21]. We restrained the frog with both hands and separated individual toes for probing. To minimize movements of the frog or operator, the dorsal side of the toe was attached carefully to a soft tube attached to a vacuum pump which held the exposed toe fixed in one position. The tube with the arrested toe pad was then positioned with help of a manual micro-manipulator underneath a two-axis force transducer (noise level in both axes ≈0.5 mN; bending stiffness in both axes approx. 108 N m^−1^). By moving the force transducer with a motorized stage controlled through a customized LabView program, we performed two sets of measurements: (i) a lateral movement to measure friction (travel of 6 mm in 10 s with a preload of 2 mN) in the proximal and distal direction of the pad to test for directionality and (ii) three detachment movements (2 mm in 10 s) to measure adhesion, namely after an initial attachment of the pad and after the two lateral movements.

Synchronously with the force data acquisition, the contact area of the pad/tubercle was recorded using a stereo microscope (Leica M80) equipped with co-axial illumination in order to yield high-contrast images [22]. The contact area was extracted at the point of the maximum force, using similar threshold routines in Matlab as described above.

Friction and adhesion were tested for each digit of the fore and hindlimbs on the distally located adhesive pads and most of the first subarticular tubercle, just proximal to the pad.

### (e) Scanning electron images of adhesive pads and tubercles

One frog was sacrificed via a lethal dose of benzocaine 250 mg l^−1^ and its fore and hindlimbs severed. After fixation of the tissue with 2.5% glutaraldehyde for 24 h, specimens were rinsed with 0.1 M phosphate-buffered sucrose followed by distilled water. The specimens were dehydrated with an alcohol series and critical point dried. Individual toes were mounted and sputter coated with gold before being viewed at 2000× and 15 000× using a Phillips SEM 500 scanning electron microscope.

## 3. Results

### (a) Ground reaction forces involved in the clamping grips

After each frog was placed carefully in a ‘head-upwards’ orientation, they either rested (27 out of 45 trials on the smooth surface and 31 out of 49 trials on the rough surface) or lowered themselves down (18 out of 45 trials on the smooth surface and 18 out of 49 trials on the rough surface; see also electronic supplementary material, video S1). The similarities between the forces from frogs holding on at rest and frogs climbing down were such that they were pooled for further analysis (Wilcoxon tests, *p* > 0.05 for all comparisons).

**Figure 2.**
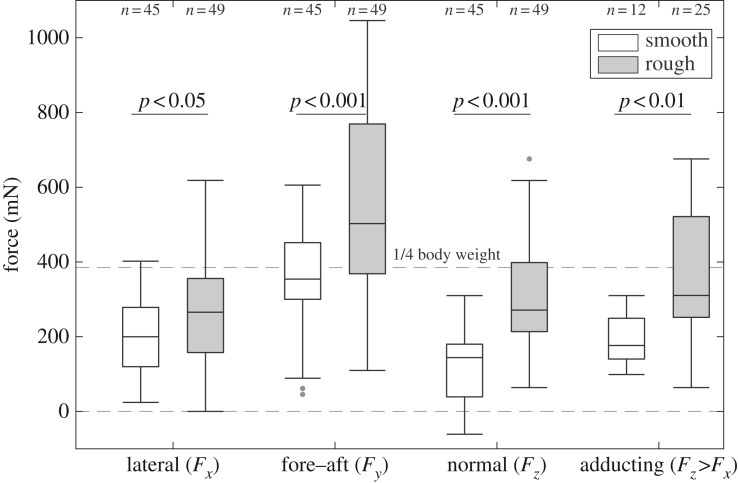
Ground reaction forces on the smooth and rough (anti-adhesive) substrates. Individual force components (*F*_*x*_, *F*_*y*_ and *F*_*z*_; mean of left and right) for the forelimbs. Clearly, distinguishable adducting forces were normal forces where *F_z_*>*F_x_* (right-hand pair of box plots).

Figure 2 shows the three force components with *F*_*x*_ being the lateral force, *F*_*y*_ the force along the gravity axis (both in the plane of the transducer platform surface) and *F*_*z*_ the normal force perpendicular to the surface. Additionally, we plotted the normal forces which were greater than the lateral forces (*F_z_* > *F_x_*). Against the smooth platform surface, the average lateral force per forelimb was 199.7 ± 165.3 mN (median ± interquartile range; given for all subsequent values, unless noted otherwise) and 144.1 ± 147.4 mN for the normal force showing that frogs stayed attached by mainly using friction forces to compensate for their tilt. The average *y*-force (along the gravity vector) per forelimb was 354.0 ± 153.1 mN, therefore compensating a little bit less than a quarter of the body weight (23% of the average body weight of 157 g).

When frogs were challenged to climb the rough surface, all force components increased significantly. The lateral force for a forelimb increased 1.3 times (265.5 ± 203.5 mN; Wilcoxon test: *R* = 1841, *z* = −2.24, *p* < 0.05), whereas compressive normal forces nearly doubled (1.9 ×; 271.2 ± 189.8 mN; Wilcoxon test: *R* = 1376, *z* = −5.76, *p* < 0.001). This indicates a stronger clamping grip when frogs could not use their adhesion pads on the anti-adhesive rough surface. Along with a stronger grip, frogs also compensated a larger amount of their body weight with the forelimbs (*y*-force: 502.8 ± 405.2 mN per forelimb, equals to about 33% of the body weight; Wilcoxon test: *R* = 1617, *z* =−3.94, *p* < 0.001).

As frogs often pulled in an oblique way at the force transducers and thus creating a normal component which could mask potential adducting forces, we considered only the cases where *F_z_* > *F_x_* which shows the adducting forces (figure 2*a*, right-hand plots); for the few cases (12 out of 45 trials for the smooth surface and 25 out of 49 trials for the rough surface), the compressive forces on the rough surface (310.4 ± 281.3 mN) were significantly larger than on the smooth surface (176.4 ± 109.1 mN; Wilcoxon test: *R* = 136, *z* = −2.97, *p* < 0.01).

### (b) Contact area in climbing frogs

Similar to the experiments on the force platform, frogs either rested (22 out of 51 trials from all six frogs) or climbed downwards (29 out of 51 trials). We did not find differences in the contact area (of the feet only, i.e. excluding the thighs and belly in contact) between resting frogs or climbing frogs (Wilcoxon test for all comparisons: *p* > 0.05) and thus pooled the data for further analysis.

When frogs climbed the flat or curved substrates, most of the adhesive pads came into surface contact. Despite the frogs having four digits on the forelimbs and five digits on the hindlimbs, the larger forelimb pads not only compensated for the missing digit but exhibited an approximately 1.5 times larger area compared to the hindlimbs (the statistical details are given in electronic supplementary material, table S2). The pad area of the forelimbs was similar on the flat and 120 mm tube but lower on the smaller 44 mm tube.

The subarticular tubercles did not differ in contact area between fore and hindlimbs and only made contact when frogs climbed curved surfaces. After having presumably reached the maximum contact area on the 120 mm tube, the area of tubercles did not increase further on the smaller tube.

**Figure 3.**
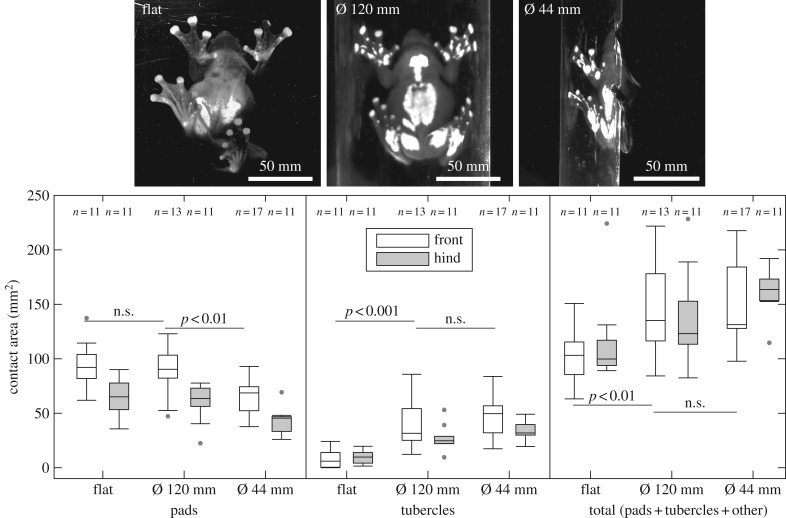
Contact area measurements. The area of pads, tubercles and the total area of fore and hind limbs (open and filled boxes, respectively; mean of left and right limbs) were recorded on a flat and two curved surfaces (cylinders with outer diameter of 44 mm and 120 mm). Images: frogs attached to the different surfaces. Note the additional subarticular tubercles and other large areas of the feet coming into contact when adhering to curved surfaces.

Frogs also increased the overall contact area of their limbs by using other parts on their feet, like parts of the palms/soles or the ventral digit skin without pads and tubercles. In particular, the hindlimbs significantly increased contact area by often involving such areas of the feet and legs (e.g. see the images in figure 3 for both curved surfaces); we excluded the thigh and belly area from our measurements as they were found in contact only when frogs rested.

### (c) Forces of individual pads and tubercles

**Figure 4.**
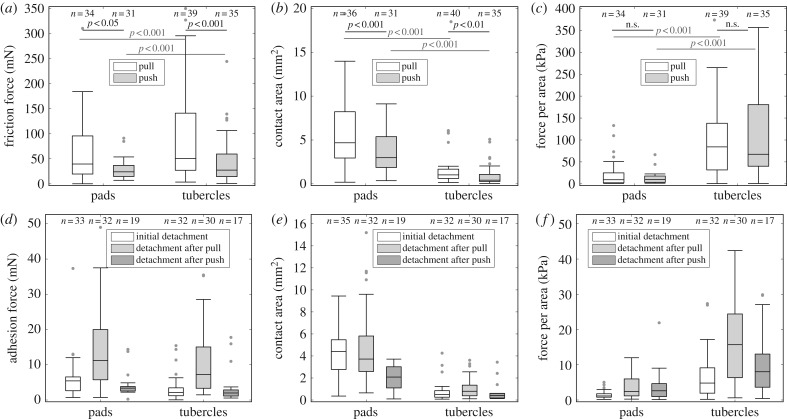
Friction and adhesion of pads and tubercles. Friction force, contact area and shear stress (*a*–*c*) were measured by a short drag of the toe along its longitudinal axis in either the proximal or distal directions (pull and push); adhesion force, contact area and adhesive stress (*d*–*f*) were measured during a perpendicular detachment before the drag (‘initial detachment’) and directly after a pull or push (‘detachment after pull’ and ‘detachment after push’, respectively).

We collected the data from all digits of fore and hindlimbs of one frog and pooled fore and hindlimbs for further analysis. Figure 4*a*–*c* show the peak friction forces, contact area and the force per area (shear stress) during a proximal pull and distal push. Friction forces for pads and tubercles were similar between each other but showed higher forces in the pulling than in the pushing direction (statistical results are listed in electronic supplementary material, table S3). Similarly, the contact area for both structures is increased during a pull. However, as tubercles were much smaller in size (figure 4*b*), they exhibited about 6.6 times higher frictional stresses than pads (figure 4*c*), supporting our idea about their possible role as frictional pads.

Adhesive forces (figure 4*d*–*f*) were roughly an order of magnitude smaller compared with friction forces. Peak adhesion forces were dependent on the time of detachment and the structure being probed (statistical results are listed in electronic supplementary material, table S4). For both structures (pads and tubercles) forces were significantly larger after a pull than before a drag or after a push. Adhesive forces were similar for pads and tubercles (detachment after a pull) but as pads were much larger in contact area (figure 4*e*), the adhesive stress was about 6.2 times lower than for the tubercles (figure 4*f*).

### (d) Morphology of adhesive pads and tubercles

**Figure 5.**
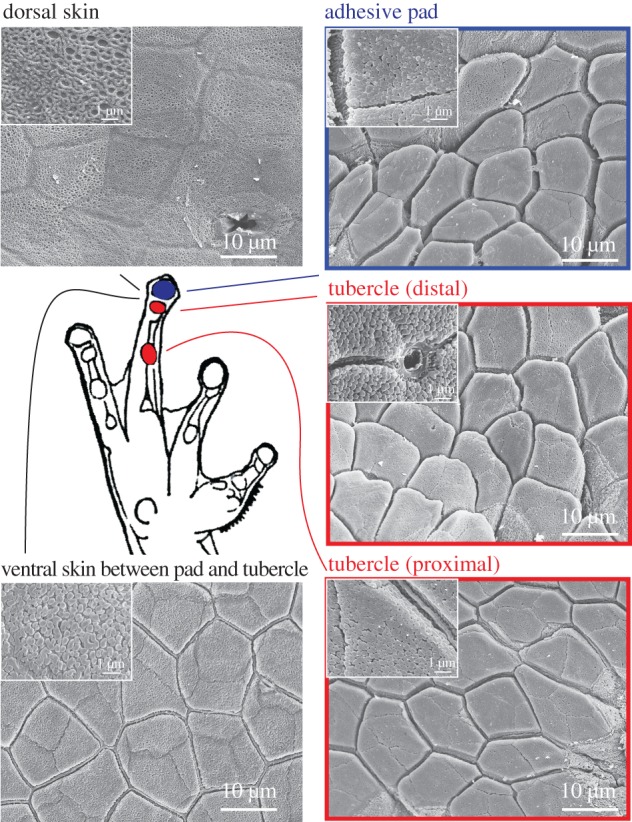
Comparative survey of pads and subarticular tubercles on a forelimb of *Rhacophorus omeimontis*. Tubercles resembled the gross morphology of adhesive pads, including the presence of nanopillars (see high magnification insets). The ventral areas around the pads and tubercles and the dorsal skin exhibited very shallow cells (sometimes only outlines) and only the ventral surfaces showed nanopillars. (Online version in colour.)

The fine structure of the toe pads of *R. dennysi* is typical of that of other rhacophorid (and also hylid) tree frogs [14,23,24]. Separated from the surrounding epithelium by circumferential and proximal grooves, the toe pad epithelium consists of flat-topped polygonal cells, separated from each other by channels (figure 5). At high magnification, it can be seen that these ‘flat tops’ actually consist of a dense array of 200–300 nm diameter nanopillars that cover the surface of each epithelial cell (figure 5, insets).

The subarticular tubercles, situated more proximally on each digit, are considerably smaller than the toe pads (indicated in schematic of figure 5). Although domed, they are not surrounded by grooves. Their fine structure is similar to that of toe pad epithelial cells, though, in the main, the channels surrounding each cell are slightly shallower than in toe pads. As in toe pads, their surface is covered in nanopillars, though at a lower density than in the toe pads. The cells that comprise the remaining ventral surface of the digits are also covered in nanopillars, though the ‘channels’ that separate them are very shallow and usually have a small ridge running along the channel centre. All these ventral structures differ in morphology from the epithelium of the dorsal surface of the digits. Dorsal epithelial cells lack both channels and nanopillars and have a spongy appearance at high magnification (figure 5).

## 4. Discussion

### (a) The use of clamping forces

As tree frogs use adhesive pads to climb even flat surfaces, it was unclear to what extent clamping forces would be used on the force measurement array. Unfortunately, our force platform did not allow us to distinguish in all cases between ‘true’ adducting forces and normal forces resulting from an oblique pull. However, the cases where normal forces were greater than the lateral forces showed a difference between the two substrates tested. In addition, the overall normal forces (i.e. all cases) increased significantly when frogs struggled to attach to non-adhesive surfaces, whereas the lateral forces did not change which points towards a clamping grip.

By clasping around the object and creating adducting forces, the friction between the limb skin surface and the substrate can increase. The clamping grip we describe here was caused by opposing limbs and not within a hand, foot or even tail as described for many other arboreal animals using prehensile grips [25]. However, in few instances, the frogs placed their feet such that neighbouring toes touched separate platforms and thus also revealed gripping forces within individual feet (see also electronic supplementary material, video S1).

On both surfaces (smooth and rough) frogs generated large shear forces by positioning their fore limbs around the structure. Against a flat platform, vertically climbing animals usually pull on the surface with their forelimbs (tensile normal forces) and push into the surface with their hind limbs (compressive normal forces) in order to compensate the pitching moment of their body (for frogs: [2]; for cockroaches: [26] and for geckos: [27]). On our octagonal platform, the tensile normal forces of the forelimbs were replaced by the lateral forces as the frogs' arms reached far enough around the structure. The friction between two hard bodies is governed by Amontons' friction law whereby the friction is proportional to the compressive (loading) force. Although adhesive pads in frogs are very soft and thus only need very little loading force for good attachment, an increase in compressive force helps friction when adhesion is compromised on a non-adhesive surface.

To the best of our knowledge, our study is the first study testing the grasping forces of climbing animals using adhesive pads. An earlier study by Manzano *et al.* [13] investigated the grasping force in frogs by pulling away a frog from a horizontally placed dowel, which does not show the clasping forces directly. Most other studies looked at grasping forces of animals attaching to flat surfaces. For example, Han *et al.* [28] measured the left–right grasping forces of locusts grasping on to a flat substrate covered in different grades of rough sandpaper. The authors showed that the grasping forces increased with increasing slope angle, highlighting how the adduction forces keep the claws engaged.

For smaller insects such as ants, most curved surfaces (tree trunks or larger branches) appear to be virtually flat. Nevertheless, a comparative study by Federle & Brüning [29] on the climbing behaviour of closely related ant species (genus *Crematogaster*) showed that two species were able to climb the (narrow) slippery stems of their host plant (*Macaranga*) by not only interlocking their fine claws with the waxy layer but by stretching their limbs further outwards and thus using adduction forces.

### (b) Contact area and function of tubercles

When frogs climbed our transparent surfaces, in all cases, the adhesive pads came into contact with the substrate. Strikingly, on curved surfaces, the frogs additionally recruited subarticular tubercles for an increased contact area. Frogs increased the contact area to the substrate further by employing ventral skin from other parts of their body too. Not only did they often use the ventral skin on their toes, lying between the pads and tubercles but sometimes even large parts of the thighs and belly. However, it was evident from a few cases in our study and other earlier studies [16,30], that frogs only use belly and thigh skin in contact when resting. During locomotion, only the feet are in contact with the substrate. We believe that the recruitment of the tubercles is a crucial part when climbing curved surfaces as they can presumably withstand a larger amount of friction (see below). However, it is worth mentioning that apart from the pads and tubercles, other ventral skin areas of the feet also bear nanopillars which are thought to promote friction forces. As we found in our study, these areas also came into contact with the surface during climbing of the cylinders.

The possible function of these tubercles as friction pads was mentioned by Noble & Jaeckle [31] and Ernst [32] and the pads' morphology was investigated in greater detail by Drotlef *et al.* [33]; however, this study is the first to address the role of tubercles for climbing curved surfaces. Herrel *et al.* [12] showed that monkey tree frogs (genus*Phyllomedusa*) managed to walk on even narrower (e.g. 1 mm and 4 mm diameters), horizontally placed tubes by wrapping individual digits around the substrate. The fact that the frog's centre of mass needs to be balanced above the substrate suggests that the skin on the digits has to compensate for potentially strong torques around a cylindrical substrate, and highlights the need for friction pads.

### (c) Force measurements on individual pads and tubercles

The force measurements on individual pads and tubercles in restrained frogs showed that tubercles exhibited higher frictional stresses than pads. This finding supports our idea that tubercles are used to enhance a friction grip. However, tubercles also showed larger *adhesive* stresses, which was surprising given that the pads are usually the primary attachment devices. We believe that the pads still perform this role as main attachment devices as they are the only structures coming into contact when the frog is climbing a flat vertical surface. Individual contact area of tubercles are about five times smaller than the average pad area (measured during our pull-off experiments), but the sum of all tubercles in contact on curved surfaces is very close to the area of the pads (figure 3). We therefore believe that tubercles are far more important than previously thought.

Our results showed only weak evidence for directionality on the pads or tubercles for friction and a small but significant increase in adhesion after a pull. Previous research [30,34] has shown a dramatic change in contact area between a pull and push for the pads which we saw in some of our trials. We believe that our method of holding the toe and pad/tubercle firmly in place prevented the buckling of the structure during a push and thus a peeling of the pad from the substrate. We believe that had we measured pads and tubercles in a ‘footloose’ condition (i.e. not immobilized), similar to the study of Bullock *et al.* [34] on insects, adhesive pads would have been more prone to buckling owing to their larger size (more liquid filled) and distal location on the toe. Otherwise, there is no obvious evidence from the topological structures of the pads or tubercles themselves which would point towards a directionality. However, a recent study by Nakano & Saino [35] showed that pads bear internal tonofilaments which are angled proximally and may cause directionality as proposed by previous studies [36,37]. We furthermore predict that tubercles would be stiffer than pads, as indicated from their smaller size and shallower channels surrounding the cells.

The chances of buckling and collapsing of the structures are less likely when the structures are subject to a pull and confirms the reorientating behaviour of frogs to climb up or down a vertical surface with their heads pointing upwards [30]. When doing so, even their hindlimbs are rotated so that all toes are orientated more or less along the gravity vector and are in pulling orientation. A similar behaviour was recorded for geckos [38]. In contrast, insects might be more restrained in this regard as only their first leg joint at the trochanter is able to rotate the leg in the dorsal–ventral plane; as a consequence, their fore legs tend to point towards the front, whereas the hindlegs point towards the back. Previous studies have shown the use of additional friction pads when insects have to use their legs for pushing [39–41]. As these frictional pads are located more proximal on the digit or the tarsus, they are less prone to buckling when the leg is subject to a pushing force and thus can maintain (or even increase) the contact area under a pushing load [42].

## 5. Conclusion

Our study demonstrated how tree frogs using adhesive pads for climbing smooth surfaces can cope with curved and anti-adhesive substrates by applying clamping grips. Such grips use not just the adhesive pads found on the distal ends of each digit but also recruit additional adhesive structures located proximally to the adhesive pads. Compared with the adhesive pads, subarticular pads can withstand higher friction forces in potentially different directions and are thus ideal for climbing narrow substrates like twigs or branches.

Understanding how animals with adhesive pads use gripping forces to aid their climbing will provide insights that will be beneficial for the development of climbing robots. The use of gripping forces on curved structures increases climbing efficiency and reduces the dependence on specialized adhesive structures. In addition, because adhesive structures often depend on shear and loading forces for effective function, gripping forces will actually aid the adhesive forces (e.g. in geckos [43] or artificial mimics [44,45]).

## Supplementary Material

S_Tab_1-4

## Data accessibility

All data are available from the Dryad Digital Repository (http://dx.doi.org/10.5061/dryad.pd7vt) [17].
