# Supplementary material for: The use of clamping grips and friction pads by tree frogs for climbing curved surfaces
Source: Proc Biol Sci. 2017 Feb 22;284(1849):20162867. doi: 10.1098/rspb.2016.2867 (PMC5326540; doi:10.1098/rspb.2016.2867)
Supplement: S_Tab_1-4 [file rspb20162867supp1.pdf]

Individual	Mass (g)	SVL (mm)	Fore limb span (mm)
1	157	112	191
2	91	95	163
3	156	119	194
4	177	127	190
5	162	106	201
6	196	121	194
Average (±SD)	157±36	113±12	189±13

S_Tab_ 1: **Mass, snout-vent length (SVL) and fore limb span for the six Chinese gliding frogs (*Rhacophorus dennysi*).**

	overall effect	
	limbs	surface
Pads	$F_{1,64} = 35.29, P < 0.001$	$F_{2,64} = 10.80, P < 0.001$
Tubercles	$F_{1,64} = 3.23, P = 0.077$	$F_{2,64} = 33.98, P < 0.001$
Total	$F_{1,64} = 0.20, P = 0.66$	$F_{2,64} = 11.64, P < 0.001$

	pairwise comparison		
	flat–120 mm	flat–44 mm	120–44 mm
Pads	$P > 0.05$	$P < 0.01$	$P < 0.01$
Tubercles	$P < 0.001$	$P < 0.001$	$P > 0.05$
Total	$P < 0.01$	$P < 0.001$	$P > 0.05$

S_Tab_ 2: **Statistical results for testing the effects of limbs (fore & hind) and surface texture (smooth & anti-adhesive) on contact area of pads, tubercles and total area.** A 2-Way ANOVA with subsequent pairwise t-tests was used. Interactions of limbs with surface were not significant for all cases. P-values were adjusted for multiple comparisons using Bonferroni correction.

**Friction** force		
Pads (pull) vs Tubs (pull)	$R = 1152, z = -1.17, N = 34, 39$	$P > 0.05$
Pads (push) vs Tubs (push)	$R = 958, z = -1.03, N = 31, 35$	$P > 0.05$
Pads (pull vs push)	$R = 305, z = 2.32, N = 34, 31$	$P = 0.04$
Tubs (pull vs push)	$R = 526, z = 3.91, N = 39, 35$	$P < 0.001$
Contact area		
Pads (pull) vs Tubs (pull)	$R = 1915, z = 5.5, N = 34, 39$	$P < 0.001$
Pads (push) vs Tubs (push)	$R = 1441, z = 5.16, N = 31, 35$	$P < 0.001$
Pads (pull vs push)	$R = 410, z = 3.65, N = 31$	$P < 0.001$
Tubs (pull vs push)	$R = 508, z = 3.16, N = 35$	$P < 0.01$
Force per area		
Pads (pull) vs Tubs (pull)	$R = 831, z = -4.72, N = 34, 39$	$P < 0.001$
Pads (push) vs Tubs (push)	$R = 672, z = -4.70, N = 31, 35$	$P < 0.001$
Pads (pull vs push)	$R = 246, z = 0.98, N = 31$	$P > 0.05$
Tubs (pull vs push)	$R = 250, z = -0.81, N = 35$	$P > 0.05$

S_Tab_ 3: **Statistical results for *friction* on individual pads and tubercles.** For testing the effects of pulling and pushing, paired Wilcoxon tests were used. To test between pads and tubercles regular Wilcoxon test were applied. P-values were adjusted for multiple comparisons using the Bonferroni correction.

**Adhesion** force		
time of detachment	$F_{2,157} = 20.66$	$P < 0.001$
structure	$F_{1,157} = 9.15$	$P < 0.01$
interactions	$F_{2,157} = 1.11$	$P > 0.05$
pads (initial vs after pull)	$R = 65, z = -3.3$	$P < 0.01$
pads (after pull vs after push)	$R = 129, z = 3.15$	$P < 0.01$
tubs (initial vs after pull)	$R = 43, z = -3.06$	$P < 0.01$
tubs (after pull vs after push)	$R = 120$	$P < 0.001$
Pads vs tubs (after pull)	$R = 1078, z = 0.98$	$P > 0.05$
Contact area		
time of detachment	$F_{2,159} = 8.27$	$P < 0.001$
structure	$F_{1,159} = 151.44$	$P < 0.001$
interactions	$F_{2,159} = 3.79$	$P < 0.05$
pads (initial vs after pull)	$R = 245, z = -0.06$	$P > 0.05$
pads (after pull vs after push)	$R = 135, z = 3.46$	$P < 0.01$
tubs (initial vs after pull)	$R = 96$	$P < 0.01$
tubs (after pull vs after push)	$R = 110, z = -1.14$	$P > 0.05$
Pads vs tubs (after pull)	$R = 1406, z = 5.6$	$P < 0.001$
Force per area		
time of detachment	$F_{2,157} = 9.42$	$P < 0.001$
structure	$F_{1,157} = 45.63$	$P < 0.001$
interactions	$F_{2,157} = 0.5$	$P > 0.05$
pads (initial vs after pull)	$R = 52, z = -3.58$	$P < 0.001$
pads (after pull vs after push)	$R = 48, z = -1.03$	$P > 0.05$
tubs (initial vs after pull)	$R = 44$	$P > 0.05$
tubs (after pull vs after push)	$R = 51, z = -2.83$	$P < 0.01$
Pads vs tubs (after pull)	$R = 702, z = -4.3$	$P < 0.001$

S_Tab_ 4: **Statistical results for *adhesion* on individual pads and tubercles.** For the tests on adhesion, the Scheirer-Ray-Hare test (extension of Kruskall-Wallis ANOVA on ranks) was used to test the two factors, namely 1) time of detachment (initial detachment, detachment after pull and detachment after push) and 2) the structures investigated, i.e. pads and tubercles. Wilcoxon tests with adjusted P-values were used for post hoc comparisons.
